# Dollar shocks and cross-border capital flows: Evidence from 33 emerging economies

**DOI:** 10.1371/journal.pone.0319570

**Published:** 2025-03-26

**Authors:** Minjie Hu, Xuemei Yuan

**Affiliations:** 1 Business School, University of Jinan, Jinan, Shandong, China; 2 Finance Research Institute, Shandong Collaborative Innovation Center for Capital Market Innovation and Development, University of Jinan, Jinan, Shandong, China; Adana Alparslan Turkes Science and Technology University: Adana Alparslan Turkes Bilim ve Teknoloji Universitesi, TÜRKIYE

## Abstract

Under the dollar-dominated international monetary system, the cross-border capital flows of emerging economies reverse sharply following policy shifts by the Fed. To investigate the sensitivity of cross-border capital inflows to dollar shocks, we analyze 33 emerging economies from 2006Q1 to 2021Q4 and use the panel quantile model to explore the dynamic evolution of dollar appreciation shocks at different stages of capital inflows, especially the tail effects. We find that dollar appreciation shocks reduce the total cross-border capital inflows of emerging economies. This impact is mainly through internal and external financial cycle difference channels. Dollar shock impacts differ significantly across different quantiles of capital inflows. Specifically, dollar appreciation shifts the capital inflow to the left and increases the severity of the left-tail risk of capital flows. More flexible exchange rate regimes exacerbate the negative effects of dollar shocks across the distribution of capital inflows. The moderating effect of the fixed exchange rate and intermediate exchange rate systems on external shocks are effective in low quantiles of capital inflows. The sensitivity of “capital flows at risk” to dollar shocks depends on national structural characteristics. As a key risk factor for emerging economies, US dollar appreciation can predict the trend of cross-border capital inflows. Countries should adopt policy measures to curb the adverse effects of US dollar fluctuations.

## 1. Introduction

Despite the complete demise of the dollar-centred Bretton Woods system, the dollar still dominates international finance and trade, prompting dollar shocks to become concomitant with global financial cycles. Federal Reserve monetary policy, US economic and financial conditions, and changes in global investor risk sentiment can all cause changes in the dollar’s movements, and these factors will also affect the global financial cycle. The importance of the dollar in global financial cycles and as a global safe asset makes the ‘anchored’ dollar more attractive [1]. With the development of global financial integration, dollar shocks are increasingly affecting the macroeconomic development of emerging economies through financial channels, and countries with higher balance sheet fragility and less credible monetary policies are more vulnerable to dollar spillovers [2]. As a barometer of global investors’ risk preferences, US dollar exchange rate volatility is considered an important risk factor for the economic growth of emerging economies [3]. In the first quarter of 2020, the dollar index increased by approximately 10%, and emerging economies’ real GDP growth was approximately -1.8%. Clearly, dollar shocks are closely linked to weaker economic prospects in emerging economies. According to the IMF’s April 2023 Global Financial Stability Report, the sudden failure of Silicon Valley Bank in the United States and the loss of confidence in Credit Suisse have reminded countries of the challenges posed by the interaction of tighter monetary and financial conditions and the accumulation of vulnerabilities. If current financial market stresses do not abate and lead to a decline in global risk-taking and a consequent reversal of cross-border capital flows, emerging economies could be exposed to greater risk. Adverse external shocks tend to drive flows into the dollar, leading to large cross-border capital outflows from emerging economies.

Cross-border capital flows in emerging economies are influenced by a combination of domestic pull factors and global push drivers, particularly the global financial cycle. Given the important role of the US dollar in the global financial cycle, more attention has been devoted to the impact of US factors such as US monetary policy [4,5] and the Federal Reserve’s balance sheet policy [6] on cross-border capital flows in emerging economies. To some extent, the trend of the US dollar reflects the expected changes in US economic growth prospects, fiscal policy changes, and monetary policy changes. Therefore, this paper places the impact of the US dollar and cross-border capital inflows of emerging economies in a unified analytical framework with broad implications and attempts to answer the following questions: How do dollar shocks affect cross-border capital flows in emerging economies? Does this effect work through the channels of internal and external financial cycle divergence? Can a floating exchange rate regime help alleviate the adverse effects of dollar shocks? How does the marginal effect of dollar shocks change at different stages of the development of capital inflows? Do structural characteristics mitigate the impact of dollar shocks on low quantiles of capital inflows?

Based on this, this paper selects data on 33 emerging economies from 2006q1 to 2021q4 and takes the dollar appreciation and cross-border capital inflow of emerging economies as examples. The comprehensive feasible generalized least squares method (FGLS) is used to study the impact of dollar shocks on the cross-border capital inflows of emerging economies and the regulatory role of the exchange rate system. Furthermore, a panel quantile model (QRPD) is used to explore the marginal effect and evolution of dollar shocks at different development stages of capital inflows. First, this paper finds that a dollar appreciation shock will lead to a decline in the total cross-border capital inflows of emerging economies, which affects cross-border capital inflows mainly through the difference between domestic and foreign financial cycles, including domestic and foreign risk premiums, asset prices, exchange rate fluctuations, and exchange rate expectations. Dollar shocks have the least adverse impact on capital inflows at fixed exchange rates. Second, the impact of a dollar shock differs significantly at different quantiles of capital inflows. A dollar appreciation shock will cause the distribution of capital inflows to shift to the left, increasing the severity of the tail risk of capital flows. When the exchange rate regime is more flexible, the adverse effects of dollar shocks are exacerbated in the entire distribution of capital inflows, while the moderating effects of fixed and intermediate exchange rate regimes on external shocks are effective at low quantiles. Third, the sensitivity of the value of capital flows at risk to dollar shocks depends on the characteristics of the country, such as the degree of financial integration, trade openness, financial development, and capital account control.

The main marginal contributions are as follows: (1) Expanding the analytical framework of the drivers of cross-border capital inflows. Most existing studies on the spillover effects of dollar shocks focus on macroeconomic fluctuations, while few studies on the drivers of cross-border capital inflows consider the impact of dollar shocks. Taking 33 emerging economies as examples, this paper analyses the impact of dollar shocks on cross-border capital inflows and compares the differences in this impact under different exchange rate regimes, providing an important supplement to the existing drivers. (2) An analytical framework is constructed to analyze the impact of a dollar shock on cross-border capital inflows from the perspective of internal and external financial cycle differences. Existing studies have not systematically analyzed the mechanism of the impact of dollar shocks on cross-border capital inflows, but the analysis from the perspective of the difference between internal and external financial cycles is helpful to more comprehensively understand the impact of dollar shocks on the cross-border capital inflows of emerging economies. (3) A panel quantile model is used to distinguish different levels of cross-border capital inflows to investigate the dynamic changes in its driving factors, focusing on the marginal effect of dollar impacts at different levels of capital inflow and the influence of country characteristics on low quantile capital inflows. Most studies have used ordinary least squares (OLS) to study the average influence of the drivers of cross-border capital flows or used the threshold method and probit model to investigate the influence of the drivers of cross-border capital flows on the probability of abnormal flows. The former does not consider the potential structural changes of capital flows, while the latter is arbitrary and cannot quantify the severity of extreme risks.

The remainder of this paper is structured as follows: The second part provides a concise literature review and research hypotheses. The third part focuses on the selection of variables and the establishment of the model framework. In the fourth part, empirical analysis and findings are presented. The fifth part highlights potential avenues for future research. Finally, the sixth part concludes the paper by summarizing key findings and discussing policy implications.

## 2. Literature review and research hypotheses

### 2.1 Literature review

#### 2.1.1 Dollar shocks and the global financial cycle and its spillover effect on emerging economies.

As the world’s most important financing, reserve, pricing, and anchoring currency, the fluctuation of the US dollar is closely related to global financial conditions. A stronger US dollar means tighter global financial conditions. On the one hand, a stronger dollar will raise the value of debt, immediately weakening the debtor’s balance sheet and causing its financial position to shrink [7]. On the other hand, investor demand for safe assets increases global risk aversion, prompting the dollar to appreciate [8]. The spillover effects of dollar shocks help explain the global financial cycle proposed by Rey [9]. Because of the global financial cycle, even emerging economies with floating exchange rates cannot use domestic monetary policy to offset adverse shocks from developed financial markets. There exists an inherent asymmetry between advanced economies, like the US, and emerging economies, where the monetary policy of the US exerts a substantial influence on the global financial cycle, while the monetary policy of emerging economies has limited impact on the global financial cycle.

The fluctuation of dollar exchange rate significantly contributes to macroeconomic fluctuations in emerging economies. An appreciation shock in the US dollar triggers adverse effects on emerging economies, resulting in a decrease in GDP, investment, and private sector credit. These outcomes contradict the predictions of the traditional Mundell-Fleming model and emphasize that the transmission of US dollar shocks to emerging economies primarily occurs via financial channels [2]. Jiang et al. [8]constructed a dollar-driven global financial cycle model that covers dollar borrowing and currency mismatch, the US external balance sheet, US low-interest rates, and excessive privilege, and the US dollar as a global risk factor, connecting these facts to explain the spillover effects of US monetary policy. The fluctuation of the broad US dollar index is a risk factor for the economic growth of emerging economies. If the US dollar index increases by 1%, growth at risk (GaR) of emerging economies will decrease by 0.6% [3]. The more dollar debt a country has, the greater the spillover effects of a dollar shock. A prior study examined the response of 26 emerging economies to the impact of dollar appreciation based on the panel local projection method, and the results showed that the impact of dollar appreciation can predict recessions in emerging economies and that the negative impact depends on the flexibility of the exchange rate system, the credibility of monetary policy, and the amount of dollar debt [7].

#### 2.2.2 Global financial cycles and cross-border capital flows in emerging economies.

After the global financial crisis, in the classic “push-pull” analytical framework, the drivers of capital flows have significantly increased, especially the global financial cycle [10,11]. In particular, the sensitivity of cross-border bond capital inflows to global risks increased significantly after the financial crisis and Taper Tantrum [12]. Different types of capital flows have different sensitivities to global financial shocks. According to Koepke [13], portfolio capital flows are the most sensitive to global drivers, followed by bank capital flows, while direct investment is not. However, Cerutti et al. [14] argue that the global financial cycle plays a limited role and note that “the influence of domestic factors on the global financial cycle is ignored”. The impact of the global financial cycle on cross-border capital flows depends on national characteristics such as institutional quality, country risk, economic fundamentals, and policy intensity [15]. The impact of the global financial cycle on cross-border capital flows is magnified by a greater level of financial account openness [16]. Moreover, there is no consensus on how the global financial cycle affects cross-border capital flows under different exchange rate regimes. On the one hand, cross-border capital flows are more sensitive to global financial conditions under the fixed exchange rate regime [17]. On the other hand, similar to the idea of the “Dilemma”, cross-border capital flows will be affected by the global financial cycle regardless of a country’s exchange rate regime [16]. A possible explanation is that the flexible exchange rate regime fails due to the large covered interest rate parity deviation under high global risk aversion [18].

#### 2.2.3 Dollar shock, cross-border capital flows from emerging economies and their “tail risks”.

As one of the important risk factors for emerging economies, the dollar exchange rate will not only affect the macroeconomic conditions of emerging economies but also affect cross-border capital flows. In addition to global risk appetite, total capital flows are also strongly negatively correlated with US dollar exchange rates and commodity prices [19]. However, when studying the sensitivity of cross-border capital flows to global factors, the literature often ignores the spillover effects of the dollar index and commodity prices. A stronger US dollar leads to wider CIP bias, which in turn leads to slower growth in US dollar-denominated cross-border bank capital flows in emerging economies [20].

Regarding cross-border capital flows, the literature is mainly based on the level of cross-border capital flows [18,21], volatility of cross-border capital flows [22] and abnormal cross-border capital flows [23,24] and discusses three aspects, among which the volatility of capital flow and abnormal cross-border capital flows can quantify cross-border capital flow risks. However, Gelos et al. in 2019 first proposed the concept of “capital flow-at-risk (CaR)”, that is, the capital flow value at a certain low quantile in the probability distribution of capital flow conditions. If the 5% quantile is selected, this indicates that there will be a 5% probability of capital flows being lower than this value. The idea is based on value-at-risk (VaR) and growth-at-risk (GaR), where the GaR framework provides information on the overall distribution of growth and the relative importance of the drivers of future GDP growth, making it possible to quantify the severity of a sharp economic slowdown. It could help policymakers address serious downside risks. Adrian et al. [25], examining the perspective of growth vulnerability based on the GaR framework, report that the low quantile of economic growth is more likely to decline with the deterioration of the financial environment, and the amplification mechanism of the financial sector leads to growth vulnerability. Based on the CaR framework, Eguren et al. [26] describe the probability distribution of capital flows in a group of emerging economies and find that the importance of push and pull factors is related to the distribution of capital flows and that the effect is strongest in the left tail. Stricter capital inflow control and macroprudential policies reduce the probability of a country experiencing a sharp capital outflow or inflow. Gelos et al. [27] discuss the influence of push and pull factors and country characteristics on the probability density distribution of future capital inflows. This method quantifies the tail risks of cross-border capital flows and can evaluate the effectiveness of policy tools in mitigating risks, laying a foundation for the risk management framework of capital flows. Chari et al. [28] use a panel quantile model to find that different global shocks have a significant impact on the tail risk of capital flows in emerging economies. The absolute value of the regression coefficient of global shocks is the largest when the quantile is 5% and the smallest when the quantile is 95%.

### 2.2 Theoretical mechanism and research hypotheses

The transmission channels of the macroeconomic spillover effect of dollar shocks on emerging economies can be divided into trade channels and financial channels [2,29]. Traditional trade channels have been weakened by the centrality of the US dollar in global trade and financing and the integration of more emerging economies into the international financial supply chain [2]. Therefore, the impact of US dollar fluctuations on the cross-border capital inflows of emerging economies comes mainly through financial channels. Considering the influence channels by which the differentiation of internal and external financial cycles affects cross-border capital flows, the differences in internal and external financial cycles are decomposed into risk premium differences, asset price differences, exchange rate fluctuations, and exchange rate expected fluctuations, where the differences in financial indicators represent the differences in internal and external financial cycles.

In terms of the risk premium mechanism, the rise of global investors’ risk sentiment towards a country reflects an increase in global investors’ investment risk expectations towards the country, in which case investors demand an excess risk premium. When the dollar index rises, global investors’ appetite for risk increases, and investors and lenders reduce risky investments in emerging economies and shift money into safe dollar assets, driving up the dollar. This drives up the excess return required for emerging economy bonds. Under flexible exchange rates, this increase in the risk premium is achieved through currency depreciation, while under pegged exchange rates, tighter monetary policy is needed to achieve this [4]. The rise of the dollar index leads to volatility in global investor risk sentiment, changes the level of the risk premium required for emerging economies, and in turn causes a large shift in capital inflows to emerging economies.

In terms of the asset price mechanism, dollar exchange rate fluctuations act on the stock prices of emerging economies through commodity prices and investor risk sentiment. A rising dollar index leads to increasing raw material prices in emerging economies, higher costs and lower yields for related companies and is thus transmitted to stock markets worldwide. Furthermore, the appreciation of the US dollar leads to the decline of asset prices in core countries and the exacerbation of the decline in asset prices of emerging economies in the presence of risk and panic, which ultimately influences the capital inflows of emerging economies.

In terms of the exchange rate mechanism, the fluctuation of the US dollar, as the international currency of exchange, will cause exchange rate changes in other currencies and changes in investors’ exchange rate expectations. On the one hand, when the dollar index rises, that is, the exchange rate of the US dollar rises relative to a basket of currencies, the local currencies of some countries pegged to the US dollar will face depreciation pressure, the purchasing power of the currency will decline, and the exchange rate of the local currency will depreciate. On the other hand, when the US dollar is in the upward cycle, investors continue to strengthen the expectation of the appreciation of the US dollar and the depreciation of the currencies of emerging economies. When the impact of dollar appreciation leads to the depreciation of or depreciation expectations for emerging economies, to obtain higher investment returns or reduce investment losses, investors allocate more funds to dollars and dollar assets, and capital flows out of emerging economies.

Based on the above analysis, the following basic hypotheses are proposed:

H1: Dollar shocks will reduce cross-border capital inflows to emerging economies.

H2: Dollar shocks will affect the cross-border capital inflows of emerging economies through internal and external financial cycle differences, including risk premium channels, asset price differences, exchange rate fluctuations, and exchange rate expectations channels.

## 3. Data selection and model construction

### 3.1 Model construction

To test the impact of US dollar shock on the cross-border capital flows of emerging economies, a fixed-effect country panel model is constructed. The specific model is as follows:


$$capitalflow_{i,t}=\alpha +{\text{β}}_{1}\text{Du}sdx_{t}+{\text{β}}_{2}{\text{Ζ}}_{t-1}+{\text{β}}_{3}{\text{X}}_{i,t-1}+Trend+\mu_{i}+\varepsilon_{i,t}$$ (1)

where *i* denotes the country, *t* denotes the quarter; $capitalflow_{i,t}$ represents the share of foreign capital inflows in GDP; $Dusdx_{t}$ is the logarithmic difference of the dollar index and the proxy scalar of the dollar shock; ${Ζ}_{t-1}$ indicates global factors, principally US BBB bond rates, global liquidity, global investor risk sentiment (VIX), and global commodity prices; ${Χ}_{i,t-1}$ denotes domestic pull factors, including the degree of financial integration, trade openness, financial development, capital account controls, economic fundamentals, and price level; Trend represents the time trend item; $\mu_{i}$ denotes the country fixed effect; $\varepsilon_{it}$ denotes the random disturbance term; and $\beta_{1}$ is the core regression coefficient, where a negative $\beta_{1}$ coefficient indicates that the rising impact of dollar appreciation will lead to a decline in cross-border capital inflows. To mitigate the endogeneity problems caused by reverse causality, all control variables are lagged by one period.

Formula (1) focuses on the mean regression of the impact of dollar shocks on cross-border capital inflows, but the mean regression cannot reflect the whole picture of the conditional distribution; that is, the impact of dollar shocks on cross-border capital inflows may be different at different development levels of capital inflows. Hence, taking inspiration from the CaR analytical framework suggested by Gelos et al. [27], this study employs a panel quantile model to examine the dynamic changes in the marginal effect of the US dollar’s influence on the cross-border capital inflow process. According to the panel quantile model of nonadditive fixed effects proposed by Powell [30], panel quantile estimation is introduced into the framework of the instrumental variable method to make the random disturbance term include fixed effects and ensure the indivisibility of the random disturbance term. Compared with the traditional panel quantile model, the advantage of the QRPD model is that the estimated coefficient is more accurate and the results are more robust. The QRPD panel quantile model is set as follows:


$$Q_{capflow_{i,t}}\left(\tau \right)=\alpha \left(\tau \right)+\beta_{1}\left(\tau \right)Dusdx_{t}+\beta_{2}\left(\tau \right){Ζ}_{t-1}+\beta_{3}\left(\tau \right)X_{i,t-1}+\varepsilon_{i,t}$$ (2)

where *τ* represents the quantile and the other identifiers are the same as in formula (1). In the QRPD model, when the quantile is τ, the regression coefficient of the core explanatory variable describes its influence on the explained variable at the loci, so we can focus on the marginal effect of dollar shocks at the extreme level of capital inflows.

To further verify whether the structural characteristics of each country can help mitigate the impact of dollar shocks on CaR, an interaction term between dollar shocks and national structural characteristics is introduced based on Equation (2). The model is set as follows:


$$\begin{array}{l} Q_{capflow_{i,t}}\left(\tau \right)=\alpha \left(\tau \right)+\beta_{1}\left(\tau \right)Dusdx_{t}+\beta_{2}\left(\tau \right){Ζ}_{t-1}+\beta_{3}\left(\tau \right)X_{i,t-1}+\beta_{4}\left(\tau \right)S_{i,t-1}+\\ \beta_{5}\left(\tau \right)Dusdx_{t}\times S_{i,t-1}+\varepsilon_{i,t} \end{array}$$ (3)

where ${\text{S}}_{i,t-1}$ is the degree of financial integration, trade openness, financial development, capital control and other structural factors. ${\text{β}}_{5}\left(\text{τ}\right)$ is the regression coefficient of the interaction term. Under the premise that ${\text{β}}_{1}\left(\text{τ}\right)$ is significantly negative, if ${\text{β}}_{5}\left(\text{τ}\right)$ is significantly positive, this indicates that this structural feature can alleviate the adverse effects of cross-border capital inflows at different quantiles. If ${\text{β}}_{5}\left(\text{τ}\right)$ is significantly negative, this indicates that the adverse impact of dollar appreciation on capital inflows is further enhanced with an increase in this interaction variable.

### 3.2 Data selection and variable description

The explained variable is cross-border capital inflows. Capital inflows include total capital inflows (FI), direct investment inflows (FDI), portfolio investment inflows (PFI), and other investment inflows (OFI). To avoid the impact of extreme observations on the results, we follow Alberola & Serena [31] and Obstfeld et al. [17] to conduct the standardized treatment of capital inflows with GDP:


$$capitalflow_{i,t}=\frac{Capitalflow_{i,t}}{GDP_{i,t}}$$ (4)

The core explanatory variable is the dollar shock. In this paper, a change in the dollar index, which measures the change in the strength of the dollar, is defined as the dollar shock, which reflects the expected change in economic growth prospects, a change in fiscal policy and a change in the monetary policy of the United States. This index was created after the disintegration of the Bretton Woods system. It is one of the most important financial indicators in the global financial market. It measures the comprehensive change in the exchange rate of the US dollar against a basket of currencies of major developed countries.

Based on the “push and pull” framework of cross-border capital flows, the control variables mainly include domestic factors and global factors. Domestic factors include (1) the degree of financial integration; (2) trade openness; (3) the level of financial development; and (4) the openness of the capital account. The Kaopen index is constructed by Chinn and Ito [32] is adopted. The higher the index is, the greater the openness of the capital account. Additional domestic factors are (5) differences in economic fundamentals, and (6) the inflation level. Global factors include (1) US triple-B bond yields; (2) global liquidity; (3) global investors’ appetite for risk; and (4) commodity prices. Comprehensively considering the availability of sample data, data from 33 emerging economies from 2006Q1 to 2021Q4 are selected. The 33 emerging economies include Albania, Argentina, Belarus, Bosnia and Herzegovina, Brazil, Chile, China, Colombia, Costa Rica, Croatia, Egypt, El Salvador, Georgia, Hungary, India, Indonesia, Kazakhstan, Kuwait, Malaysia, Mexico, Morocco, Macedonia, Panama, Peru, Philippines, Poland, Romania, Russian Federation, Saudi Arabia, South Africa, Thailand, Turkey, Ukraine.

The selection of the main variables and data sources is shown in Table 1. Among them, the sample countries’ total U.S. dollar-denominated GDP in 2021 covers approximately 75% of the total GDP of emerging and developing economies.

**Table 1:** Variable selection and description.

Variable	Variable name	Variable Description	Data source
Gross capital inflows	FI	The ratio of total capital inflows to GDP	International Monetary Fund’s International Financial Statistics (IMF-IFS)
Direct investment inflows	FDI	The ratio of direct investment inflows to GDP	
Portfolio inflows	PFI	The ratio of portfolio investment inflows to GDP	
Other investment inflows	OFI	The ratio of other investment inflows to GDP	
Dollar (appreciation) shocks	Usdx	Dollar index is taken in logarithms	Federal Reserve website
	Dusdx	The dollar index is log-differenced	
Financial integration	Fin-Integration	Total foreign assets and liabilities as a share of GDP	IMF-IFS, authors’ calculations
Trade openness	Trade open	The ratio of total imports and exports to GDP of each country	IMF-IFS, authors’ calculations
Financial development	FD	FD	IMF-IFS
Capital account opening	Kaopen	Kaopen	Chinn and Ito [32]
Economic fundamental differences	GDP-diff	GDP growth in each country minus US GDP growth	IMF-IFS, CEIC, authors’ calculations
Inflation	CPI	The CPI index of each country is taken in logarithm	IMF-IFS, CEIC
U.S. BBB-rated corporate bond yields	BBB-yield	Detrended by HP filter castration	Federal Reserve website
Global liquidity	Usm2	The Fed M2 aggregate is taken in logarithm	Federal Reserve website
Global investors’ appetite for risk	VIX	The S&P 500 volatility is taken in logarithms	CEIC
Commodity prices	Commodity	The commodity price is taken in logarithms	WIND

To alleviate the influence of extreme values on the regression results, all continuous variables were reduced by 1%. Table 2 shows the descriptive series of the main variables. Since this data set belongs to a long panel, considering the influence of “intergroup heteroscedasticity”, “intragroup autocorrelation” and “intergroup coincident correlation” on the estimation results, corresponding tests were conducted, and the results showed that the perturbation term exhibited intergroup heteroscedasticity. Therefore, comprehensive FGLS is selected for estimation.

**Table 2:** Descriptive analysis of variables.

Variable	Variable name	Obs	Mean	SD	Min	Median	Max
Explained variable	FI	2112	0.0677	0.0826	-0.1387	0.0556	0.4056
	FDI	2112	0.0375	0.0418	-0.0617	0.0277	0.2385
	PFI	2112	0.0117	0.0342	-0.0844	0.0038	0.1436
	OFI	2112	0.0175	0.0498	-0.1193	0.0114	0.2057
Core explanatory variable	Usdx	2112	4.6213	0.1020	4.4638	4.5950	4.8029
	Dusdx	2079	0.0022	0.0266	-0.0414	0.0052	0.1127
Control variables	Fin-Integration	2112	5.7662	4.1566	0.7525	5.2389	23.5845
	Trade open	2112	0.6687	0.3521	0.1912	0.5972	1.9426
	FD	2112	0.3877	0.1428	0.1504	0.3749	0.7196
	Kaopen	2112	0.5095	0.3128	0.0000	0.4470	1.0000
	GDP-diff	2112	0.0213	0.0764	-0.2011	0.0245	0.2104
	CPI	2112	4.7506	0.3157	4.1060	4.7007	6.1290
	BBB-yield	2112	0.0000	0.7670	-0.7815	-0.1896	3.3211
	Usm2	2112	9.3118	0.3088	8.8157	9.3139	9.9677
	VIX	2112	2.9036	0.3475	2.3329	2.8445	4.0705
	Commodity	2112	5.4969	0.2783	4.8483	5.5515	6.0528

Note: Some data are missing, monthly or annual data are used to transform the balanced panel data.

## 4. Empirical analysis

### 4.1 Baseline regression: average impact of dollar shocks on capital inflows

Table 3 provides an important information reference for revealing the impact of dollar shocks on the cross-border capital inflows of emerging economies. In Table 2, the first four columns control for national fixed effects, the last four columns add the time trend item on this basis, and the two are mutually robust tests. Column (5) shows that the regression coefficient of dollar shocks on total capital inflows is negative and significant at the 1% level, indicating that a dollar appreciation shock reduces total capital inflow, which is consistent with theoretical expectations. Hypothesis 1 has been preliminarily verified. Column (6) shows that the regression coefficient of the impact of dollar appreciation on direct investment inflows is negative but not significant. A possible explanation is that domestic pull factors such as favourable institutional quality and macroeconomic environment are more critical to FDI inflows, which also suggests that global factors only play a role for individual types of cross-border capital inflows in emerging economies, similar to the conclusions of Cerutti et al. (2019). Columns (7) and (8) show that the regression coefficient of the dollar shock is significantly negative at the 1% level, indicating that a dollar appreciation shock will inhibit the inflow of securities investment and other investment inflows. Compared with the inflow of direct investment, PFI and OFI are more sensitive to the impact of dollar appreciation, which is related to their short-term and high volatility.

**Table 3:** Dollar shocks and cross-border capital inflows: Baseline regression analysis.

Variable name	(1)	(2)	(3)	(4)	(5)	(6)	(7)	(8)
	FI	FDI	PFI	OFI	FI	FDI	PFI	OFI
Dusdx	−0.4287***	−0.0138	−0.1386***	−0.2009***	−0.4166***	0.0002	−0.1478***	−0.1919***
	(0.0415)	(0.0160)	(0.0191)	(0.0275)	(0.0435)	(0.0168)	(0.0201)	(0.0290)
lfinintegration	−0.0005	−0.0000	−0.0001	−0.0000	−0.0005	−0.0000	−0.0001	−0.0000
	(0.0007)	(0.0003)	(0.0003)	(0.0005)	(0.0007)	(0.0003)	(0.0003)	(0.0005)
ltradeopen	0.0108	0.0283***	−0.0042	−0.0112	0.0100	0.0282***	−0.0042	−0.0118 *
	(0.0108)	(0.0043)	(0.0049)	(0.0071)	(0.0108)	(0.0043)	(0.0049)	(0.0071)
lFD	−0.0172	0.0113	0.0093	−0.0094	−0.0142	0.0117	0.0079	−0.0055
	(0.0353)	(0.0126)	(0.0152)	(0.0238)	(0.0355)	(0.0126)	(0.0153)	(0.0241)
lzkaopen	−0.0051	0.0036	−0.0014	−0.0057	−0.0047	0.0043	−0.0012	−0.0058
	(0.0089)	(0.0032)	(0.0037)	(0.0057)	(0.0089)	(0.0032)	(0.0038)	(0.0057)
lgdpdiff	0.0068	0.0125**	−0.0085	0.0085	0.0061	0.0121**	−0.0084	0.0082
	(0.0146)	(0.0056)	(0.0061)	(0.0104)	(0.0147)	(0.0056)	(0.0061)	(0.0105)
llcpi	−0.0266***	−0.0033	−0.0040	−0.0146***	−0.0245***	−0.0025	−0.0048	−0.0132***
	(0.0066)	(0.0023)	(0.0030)	(0.0045)	(0.0067)	(0.0023)	(0.0031)	(0.0046)
lbbbyield	−0.0064***	−0.0003	−0.0018**	−0.0023**	−0.0062***	−0.0001	−0.0020**	−0.0022 *
	(0.0017)	(0.0007)	(0.0008)	(0.0011)	(0.0017)	(0.0007)	(0.0008)	(0.0011)
lusm2	0.0173 *	−0.0111***	0.0004	0.0240***	0.0507 *	0.0164	−0.0164	0.0453**
	(0.0091)	(0.0035)	(0.0040)	(0.0061)	(0.0271)	(0.0105)	(0.0125)	(0.0180)
llvix	−0.0060 *	−0.0017	0.0004	−0.0037	−0.0078**	−0.0033**	0.0013	−0.0048**
	(0.0034)	(0.0013)	(0.0016)	(0.0022)	(0.0037)	(0.0014)	(0.0017)	(0.0024)
llcommodity	0.0221***	−0.0028	−0.0020	0.0261***	0.0185**	−0.0061 *	−0.0002	0.0239***
	(0.0081)	(0.0031)	(0.0037)	(0.0053)	(0.0085)	(0.0033)	(0.0040)	(0.0057)
Trend					−0.0006	−0.0005***	0.0003	−0.0004
					(0.0005)	(0.0002)	(0.0002)	(0.0003)
Constant	−0.0220	0.2019***	0.0345	−0.2517***	−0.1783	0.0778	0.1143	−0.3526***
	(0.1053)	(0.0406)	(0.0479)	(0.0700)	(0.1604)	(0.0618)	(0.0738)	(0.1068)
Country FE	Yes	Yes	Yes	Yes	Yes	Yes	Yes	Yes
Heteroscedasticity	Yes	Yes	Yes	Yes	Yes	Yes	Yes	Yes
Observations	2079	2079	2079	2079	2079	2079	2079	2079

Note: There are 64 periods in the original quarterly data for each country. The control variables in the regression are lagged by one period, and the sample value in the actual regression is 63 periods. ***, ** and *  denote significance at the 1%, 5% and 10% levels, respectively. Standard errors are reported in parentheses.

### 4.2 Mechanism test: internal and external financial cycle differences

The benchmark regression verifies the negative impact of US dollar appreciation on cross-border capital inflows. Does a dollar shock affect cross-border capital inflows through the difference between internal and external financial cycles? According to theoretical mechanism analysis, the difference between internal and external financial cycles can be decomposed into a risk premium mechanism, asset price differential mechanism, and the exchange rate fluctuation and exchange rate expected fluctuation mechanism. In this paper, a step-up regression method was used to test the mechanism. Table 4 reports the results of the mechanism test.

**Table 4:** Dollar shocks and cross-border capital inflows: Mechanism analysis.

Variable name	(1)	(2)	(3)	(4)	(5)	(6)	(7)	(8)
	premium	FI	price	FI	exdiff	FI	exyuqi	FI
Dusdx	−3.7798***	−0.3759***	−0.4808***	−0.4282***	−0.0833***	−0.3484***	−13.5618***	−0.4123***
	(0.6769)	(0.0477)	(0.0773)	(0.0476)	(0.0051)	(0.0462)	(2.3932)	(0.0621)
premium		0.0033***						
		(0.0012)						
price				0.0365***				
				(0.0131)				
exdiff						0.3871***		
						(0.0940)		
exyuqi								0.0015***
								(0.0005)
Trend	Yes	Yes	Yes	Yes	Yes	Yes	Yes	Yes
Controls	Yes	Yes	Yes	Yes	Yes	Yes	Yes	Yes
Country FE	Yes	Yes	Yes	Yes	Yes	Yes	Yes	Yes
Heteroskedasticity	Yes	Yes	Yes	Yes	Yes	Yes	Yes	Yes
Observations	1431	1431	1754	1754	2079	2079	1197	1197

Note: Country-level money market rates, stock market indices and real effective exchange rates are unbalanced panel data. Therefore, there are missing values in the data on internal and external financial cycle differential mechanisms such as the risk premium mechanism, asset price mechanism and exchange rate expectation mechanism. ***, ** and *  denote significance at the 1%, 5% and 10% levels, respectively. Standard errors are reported in parentheses.

First, to verify the existence of the channel by which the dollar shock affects cross-border capital inflows by influencing the change in the risk premium, the difference between the change in the policy interest rates of various countries and the change in the shadow interest rates of the United States is selected as the proxy variable for the risk premium mechanism. Column 1 of Table 4 reports the regression results of the impact of risk premium changes on the US dollar. The results show that the coefficient of the dollar shock is negative and significant at the 1% level, indicating that the dollar shock has a significant negative effect on the change in the risk premium. This may be because the sensitivity of the U.S. shadow interest rate to a U.S. dollar shock is greater than the sensitivity of policy rates in emerging economies, meaning that a rise in the U.S. dollar index will lead to a greater rise in U.S. policy rates than in policy rates in emerging economies; thus, the risk premiums in emerging economies are relatively reduced. When the level of interest rates in emerging economies is low relative to U.S. rates, global investors turn to safe assets such as U.S. bonds and arbitrage capital flows out of emerging economies (Column (2)). That is, an appreciating dollar will substantially raise the U.S. policy rate, relatively reducing the attractiveness of policy rates in emerging economies, thereby lowering domestic and foreign risk premia and decreasing cross-border capital inflows to emerging economies. The risk premium channel in Hypothesis 2 is verified.

Second, to test the existence of a channel through which dollar shocks affect cross-border capital inflows by influencing asset price movements, this paper selects the difference between the movements of national stock indices and the movements of the U.S. Dow Jones Index as a proxy for the asset price mechanism (mainly stock index movements). Column 3 of Table 4 shows the results of regressions of asset price changes on dollar shocks. The results show that the regression coefficient of the dollar shock on the variance of stock index changes is negative and significant at the 1% level, indicating that the appreciation of the dollar significantly reduces the variance of asset price changes. When the dollar index rises, global investor risk sentiment rises, U.S. asset price levels fall and cause more severe reductions in asset levels in emerging economies in response to risk and panic sentiment, thus reducing the difference between internal and external asset prices. When the difference in stock index returns narrows, investors have to take higher speculative risks in emerging economies, which in turn leads to hedged capital inflows to developed economies and a decline in capital inflows to emerging economies. The above analysis verifies the existence of the asset price channel in hypothesis 2.

Finally, both exchange rate volatility and exchange rate expectations are considered using the changes in national currency exchange rates against the U.S. dollar and the real effective exchange rate after HP filter deconversion, respectively. In this case, after removing the trend term by the HP filtering method for each country, if this value is high, this indicates that the real effective exchange rate deviates from the trend at a high level and there is an expected appreciation. Columns (5) and (7) show the regression results of exchange rate changes and exchange rate expectations on dollar shocks. The results indicate that the regression coefficient of the dollar shock is significantly negative at the 1% level, suggesting that a rise in the dollar index not only leads to exchange rate depreciation in emerging economies but also leads to investors expecting depreciation in their financial assets. Specifically, a rising dollar index, i.e., an appreciating dollar, will directly lead to a relative depreciation of emerging economy currencies under an international monetary system in which the dollar is the dominant currency, and investors will strengthen their expectations of an appreciating dollar, which in turn will generate expectations of a depreciation of emerging economy currencies. When the currencies of emerging economies depreciate or there are expectations of depreciation, investors prefer dollar-denominated assets and hedge capital flows out of emerging economies. Overall, dollar shocks, through exchange rate changes or exchange rate expectations, will in turn affect cross-border capital inflows, as evidenced by the exchange rate changes and exchange rate expectations channels in Hypothesis 2.

### 4.3 Heterogeneity analysis: the impact of exchange rate regimes

The benchmark regressions indicate that cross-border capital inflows to emerging economies are closely related to the dollar index, but does the impact of the dollar index on cross-border capital flows to emerging markets differ by exchange rate regime? There are two main types of exchange rate regimes: first, the nominal or legal (de jure) classification, which is the Annual Report on Exchange Arrangements and Exchange Restrictions (AREAER) classification prepared by the International Monetary Fund based on the exchange rate regimes declared by each member country. The second is the de facto classification, which is compiled by scholars based on the actual behaviour of individual economies. Referring to the six coarse factual classifications distinguished by Ilzetzki et al. [33], they are factual pegging, creeping pegging, managed float, free float, free fall, and dual-track exchange rate and are assigned values 1-6 in that order. The sample selected for this paper does not include countries with dual-track exchange rate regimes, some of which practised free devaluation during certain periods and are classified as free floating in this paper. Therefore, the exchange rate regimes are classified as de facto pegged, crawl pegged, managed float, and free-floating exchange rate regimes, assigned values 1-4 in that order. To make the model more concise, the exchange rate regimes are treated as categorical variables, generating two dummy variables, Reg1 and Reg2, representing fixed and intermediate exchange rates, respectively, where Reg1 takes value 1 when the exchange rate regime is 1 and 0 otherwise, and Reg2 takes value 1 when the exchange rate regime is 2 or 3 and 0 otherwise.

Table 5 presents the impact of dollar shocks on cross-border capital inflows with changes in the exchange rate regime. Columns (1) to (4) show the regression results of dollar shocks on aggregate inflows, short-term capital inflows, direct investment inflows, portfolio investment inflows, and other investment inflows, respectively, and Dusdx×reg1 and Dusdx×reg2 represent the interaction terms of dollar shocks with fixed and intermediate exchange rates, respectively, with floating exchange rates as the benchmark group. First, Column (1) shows that the regression coefficient of dollar shocks is significantly negative and significant at the 1% level, indicating that the floating exchange rate system does not insulate the total capital inflow from dollar shocks. The Dusdx×reg1 coefficient of the interaction term between the dollar shock and the fixed exchange rate is significantly positive, indicating that the sensitivity of total capital inflows to the dollar shock under the fixed exchange rate system is less than that under the floating exchange rate system. Specifically, compared with the floating exchange rate system, the fixed exchange rate can mitigate the negative impact of a dollar shock on total cross-border capital inflows and short-term capital inflows. However, the regression coefficient of Dusdx×reg2, the interaction term between the dollar shock and the intermediate exchange rate, is not significant, which means that there is no significant difference between the intermediate exchange rate and the floating exchange rate. Second, Column (2) reports the regression result of the impact of a dollar shock on FDI inflow under different exchange rate systems. The result shows that the regression coefficient of the dollar shock on FDI is negative but not significant, which further verifies the result of the benchmark regression. Column (3) indicates that the coefficient of the dollar shock is significantly negative at the 1% level, indicating that the floating exchange rate fails to moderate the dollar shock. Additionally, the regression coefficient of Dusdx×reg1 is positive, indicating that the adverse impact of a dollar shock under the fixed exchange rate regime is less than that under the floating exchange rate regime. Specifically, when the dollar shock increases by 1 standard deviation (0.027), securities capital inflows decrease by 0.0083 percentage points less under the fixed exchange rate system than under the floating exchange rate system. According to the regression results in Column (4), the regression coefficient of dollar shocks is significantly negative at the 5% level, indicating that the floating exchange rate system cannot isolate the negative impact of dollar shocks on other investment inflows. However, the regression coefficients of Dusdx×reg1 and Dusdx×reg2 are not significant, indicating that the sensitivity of other investment inflows to the impact of the US dollar has no significant difference under different exchange rate systems. In conclusion, a more flexible exchange rate system may not reduce the negative impact of the dollar’s impact on cross-border capital inflow, but the negative impact can be alleviated to some extent under the fixed exchange rate system.

**Table 5:** Dollar shocks and cross-border capital inflows: Effects of exchange rate regimes.

Variable name	(1)	(2)	(3)	(4)
	FI	FDI	PFI	OFI
Dusdx	−0.5905***	−0.0663	−0.3469***	−0.1550 *
	(0.1317)	(0.0531)	(0.0779)	(0.0823)
Dusdx×reg1	0.2604 *	0.0567	0.3069***	−0.0890
	(0.1481)	(0.0585)	(0.0799)	(0.0945)
Dusdx×reg2	0.1585	0.0825	0.0992	−0.0239
	(0.1367)	(0.0548)	(0.0815)	(0.0858)
regime1	0.0289***	−0.0023	0.0066	0.0147**
	(0.0091)	(0.0039)	(0.0044)	(0.0061)
regime2	−0.0020	−0.0029 *	0.0048 *	−0.0069**
	(0.0046)	(0.0016)	(0.0027)	(0.0031)
Controls	Yes	Yes	Yes	Yes
Trend	Yes	Yes	Yes	Yes
Country FE	Yes	Yes	Yes	Yes
Heteroskedasticity	Yes	Yes	Yes	Yes
Observations	Yes	Yes	Yes	Yes

Note: Because the exchange rate regime is a categorical variable and relatively stable, no one-period lag is performed. ***, ** and *  denote significance at the 1%, 5% and 10% levels, respectively. Standard errors are reported in parentheses.

### 4.4 Robustness test

To ensure the robustness of the baseline regression results, we considered an alternative lag period for the core explanatory variable, replacing the measure of the explained variable, replacing the regression model and eliminating sample countries. Since the benchmark regression shows that direct investment is not sensitive to the dollar shock, we focus on portfolio investment and other investment inflows and treat their sum as short-term capital inflow (SFI) to verify the robustness of dollar shocks reducing capital inflows. Table 6 reports a robustness test of the impact of dollar shocks on aggregate inflows and short-term capital inflows. First, because the impact of US dollar appreciation on the cross-border capital inflows of emerging economies may have a time lag, the core explanatory variable of US dollar appreciation impact is delayed by one period. The regression results in Columns (1) and (2) show that the regression coefficient of dollar shock after a one-period lag is significantly negative at the 1% level, which is consistent with the results of the benchmark regression. Second, following Davis et al. (2021), the scale of cross-border capital inflows divided by the sum of external assets and liabilities of each country is used as an explanatory variable. The regression results are shown in Columns (3) and (4). After replacing the capital inflow measure index, the regression results are still robust. Third, as the capital inflow of the current period may affect the capital inflow of the next period, the lagged value of capital inflow is added to the benchmark regression, and a dynamic panel model is constructed for estimation. To solve the endogeneity problem caused by the introduction of the explained variable in the long panel data with a one-period lag, the bias correction LSDV method is used for estimation. According to Columns (5) and (6), in the regression results with the introduction of cross-border capital inflows with a lag of one period, the regression coefficient of the dollar impact is still significantly negative, indicating that the dollar impact reduces cross-border capital inflows and that the result is stable. Fourth, China, Brazil, India, Russia and India are excluded from the sample given their central positions among emerging economies, and Columns (7) and (8) show the regression results. After excluding the BRICS, the regression coefficient of the dollar impact remains significantly negative at the 1% level, and the result remains robust. The above estimation results are consistent with the baseline regression results, which further verifies the robustness of Hypothesis 1.

**Table 6:** Dollar shocks and cross-border capital inflows: Robustness test.

Variable Name	(1)	(2)	(3)	(4)	(5)	(6)	(7)	(8)
	Dollar shocks lag 1 period		Replace the capital inflow measure		Dynamic panel model		Eliminating the BRICS	
	FI	SFI	FI	SFI	FI	SFI	FI	SFI
Dusdx	−0.0991**	−0.1233***	−0.1305***	−0.1132***	−0.3259***	−0.3628***	−0.3312***	−0.3467***
	(0.0126)	(0.0104)	(0.0126)	(0.0104)	(0.0773)	(0.0596)	(0.0544)	(0.0457)
L.y					0.2360***	0.2196***		
					(0.0207)	(0.0210)		
Controls	Yes	Yes	Yes	Yes	Yes	Yes	Yes	Yes
Trend	Yes	Yes	Yes	Yes	Yes	Yes	Yes	Yes
Country FE	Yes	Yes	Yes	Yes			Yes	Yes
Heteroscedasticity	Yes	Yes	Yes	Yes	Yes	Yes	Yes	Yes
Observations	2046	2046	2079	2079	2079	2079	1764	1764

Note: L.y indicates that total inflows and short-term inflows lagged by one period. ***, ** and *  denote significance at the 1%, 5% and 10% levels, respectively. Standard errors are reported in parentheses.

### 4.5 Endogeneity analysis

The endogeneity problem refers to the correlation between the explanatory variables and the error term, including omitted variables, bidirectional causality and measurement error. In this paper, there may be two-way causality between the dollar index and cross-border capital flows of emerging economies. On the one hand, changes in the dollar index affect cross-border capital flows of emerging economies; on the other hand, changes in cross-border capital flows of emerging economies may in turn affect the dollar index. To address this issue, we use the lagged one-period of dollar shocks as an instrumental variable. The instrumental variable needs to fulfill two conditions: first, it is highly correlated with the endogenous explanatory variable (dollar shock); second, it is uncorrelated with the error term. The one-period lag of the dollar shock satisfies these conditions. In terms of correlation, the dollar index itself has strong time-series correlation, and the dollar shock lagged one period is correlated with the current dollar shock. In terms of exogeneity, the dollar index in the lagged period has already been formed and will not be affected by cross-border capital flows from emerging economies in the current period, and is therefore uncorrelated with the error term.

The results of the two-stage least squares estimation by using the lagged one period of the dollar shock as an instrumental variable are shown in Table 7. Column (1) shows the results of the first section of the stage regression, where the instrumental variable has a positive and significant effect on the core explanatory variables and there is no weak instrumental variable problem (C-D Wald F statistic >  10). Column (2) presents the results of the second stage regression, which show that after mitigating endogeneity using the instrumental variable approach, the dollar shock remains negatively significant on aggregate cross-border asset inflows.

**Table 7:** Endogeneity analysis.

Variable Name	(1)	(2)
	IV1	IV2
	Ldusdx	FI
Dusdx	0.3486***	−0.5885***
	(0.0206)	(0.1923)
Controls	Yes	Yes
Country FE	Yes	Yes
C-D Wald F	278.73	
N	2046	2046

Note: ***, ** and *  denote significance at the 1%, 5% and 10% levels, respectively. Standard errors are reported in parentheses.

## 5. Further research

### 5.1 Marginal effects of dollar shocks at different levels of capital inflows

The benchmark regression examines the average impact of US dollar shocks on cross-border capital inflows, and on this basis, a panel quantile model is used to study the impact of US dollar shocks on different levels of capital flows and their conditional distributions in emerging economies, especially the tail of capital flow distribution. Columns (1) - (11) show the regression results of 11 quantiles of capital inflow, including 5%, 10%, 20%, 30%, 40%, 50%, 60%, 70%, 80%, 90% and 95%, respectively. According to Table 8, the regression coefficient of the US dollar shock is significantly negative at the 1% level, further verifying the negative impact of the US dollar shock on cross-border capital inflows and indicating that a US dollar appreciation shock leads to smaller values of different quantiles of capital inflow; that is, a US dollar appreciation shock will shift the entire capital inflow distribution to the left. Columns (1) - (11) show that the regression coefficients of the US dollar shock are significantly negative at the 5% and 10% quantiles (left tail) and the 95% and 90% quantiles (right tail). This means that a dollar appreciation shock not only reduces the value of different quantiles of the total inflow but also significantly reduces the value of the lower and upper quantiles of the total inflow, with a milder impact on the centre of the distribution. Specifically, a dollar appreciation shock greatly increases the severity of sudden stops in capital flows and decreases the severity of surges in capital flows. A possible explanation is that during periods of sudden stops in capital inflows, the impact of dollar appreciation greatly inhibits cross-border capital inflows by increasing the risk sentiment of global investors and reducing the attractiveness of financial assets in emerging economies.

**Table 8:** Panel quantile regressions: Dollar shocks and gross cross-border capital inflows.

Variable name	(1)	(2)	(3)	(4)	(5)	(6)	(7)	(8)	(9)	(10)	(11)
	5	10	20	30	40	50	60	70	80	90	95
Dusdx	−0.6632***	−0.4852***	−0.4215***	−0.4291***	−0.3486***	−0.3105***	−0.3082***	−0.4239***	−0.3262***	−0.3400***	−0.3927***
	(0.0002)	(0.0005)	(0.0041)	(0.0002)	(0.0001)	(0.0001)	(0.0001)	(0.0001)	(0.0034)	(0.0002)	(0.0003)
lfinintegration	−0.0040***	−0.0030***	−0.0008***	0.0018***	0.0034***	0.0058***	0.0062***	0.0071***	0.0098***	0.0132***	0.0135***
	(0.0000)	(0.0000)	(0.0001)	(0.0000)	(0.0000)	(0.0000)	(0.0000)	(0.0000)	(0.0000)	(0.0000)	(0.0000)
ltradeopen	−0.0473***	−0.0390***	−0.0133***	−0.0153***	−0.0073***	−0.0033***	0.0101***	0.0253***	0.0427***	0.0609***	0.0717***
	(0.0001)	(0.0000)	(0.0003)	(0.0000)	(0.0000)	(0.0000)	(0.0000)	(0.0000)	(0.0003)	(0.0000)	(0.0000)
lFD	0.0099***	0.0070***	−0.0144***	−0.0351***	−0.0406***	−0.0459***	−0.0587***	−0.0684***	−0.1232***	−0.1232***	−0.1604***
	(0.0001)	(0.0001)	(0.0007)	(0.0000)	(0.0000)	(0.0000)	(0.0000)	(0.0001)	(0.0006)	(0.0000)	(0.0000)
lzkaopen	−0.0183***	−0.0083***	−0.0183***	−0.0062***	−0.0036***	0.0041***	0.0002***	0.0042***	−0.0052***	−0.0007***	−0.0098***
	(0.0001)	(0.0000)	(0.0005)	(0.0000)	(0.0000)	(0.0000)	(0.0000)	(0.0000)	(0.0002)	(0.0000)	(0.0000)
lgdpdiff	−0.0333***	0.0029***	0.0061***	−0.0076***	0.0185***	0.0255***	0.0267***	0.0586***	0.0756***	0.0854***	0.0768***
	(0.0001)	(0.0001)	(0.0011)	(0.0001)	(0.0000)	(0.0000)	(0.0001)	(0.0001)	(0.0001)	(0.0001)	(0.0000)
llcpi	−0.0145***	−0.0150***	−0.0163***	−0.0144***	−0.0148***	−0.0142***	−0.0147***	−0.0167***	−0.0278***	−0.0256***	−0.0402***
	(0.0001)	(0.0000)	(0.0005)	(0.0000)	(0.0000)	(0.0000)	(0.0000)	(0.0000)	(0.0004)	(0.0000)	(0.0000)
lbbbyield	−0.0035***	−0.0081***	−0.0075***	−0.0074***	−0.0084***	−0.0084***	−0.0083***	−0.0112***	−0.0186***	−0.0162***	−0.0166***
	(0.0000)	(0.0000)	(0.0003)	(0.0000)	(0.0000)	(0.0000)	(0.0000)	(0.0000)	(0.0001)	(0.0000)	(0.0000)
lusm2	0.0405***	0.0187***	0.0040***	−0.0012***	−0.0102***	−0.0277***	−0.0186***	−0.0285***	−0.0563***	−0.0548***	−0.0469***
	(0.0001)	(0.0001)	(0.0007)	(0.0000)	(0.0000)	(0.0000)	(0.0001)	(0.0000)	(0.0001)	(0.0000)	(0.0000)
llvix	−0.0172***	−0.0106***	−0.0094***	−0.0024***	−0.0003***	−0.0029***	0.0001***	0.0068***	0.0149***	0.0179***	0.0125***
	(0.0001)	(0.0000)	(0.0006)	(0.0000)	(0.0000)	(0.0000)	(0.0000)	(0.0000)	(0.0002)	(0.0000)	(0.0000)
llcommodity	0.0367***	0.0231***	0.0189***	0.0189***	0.0177***	0.0152***	0.0238***	0.0189***	−0.0018***	0.0243***	0.0244***
	(0.0001)	(0.0001)	(0.0006)	(0.0001)	(0.0000)	(0.0000)	(0.0000)	(0.0001)	(0.0007)	(0.0000)	(0.0000)
Observations	2079	2079	2079	2079	2079	2079	2079	2079	2079	2079	2079

Note: The QRPD method does not yield a constant term. ***, ** and *  denote significance at the 1%, 5% and 10% levels, respectively. Standard errors are reported in parentheses.

The above results show that the impact of a US dollar appreciation shock on the cross-border capital inflows of emerging economies is dynamic in different stages of cross-border capital inflows, and has a greater dampening effect in the left tail of cross-border capital inflows. That is, the sensitivity of cross-border capital inflows to a US dollar appreciation shock is heterogeneous at different quantiles, which also proves the effectiveness and advantage of quantile regression. On the one hand, the difference between the coefficient of US dollar shocks in the tail of capital inflows and the benchmark regression coefficient indicates that the model based on the mean value of capital inflows ignores the dynamic change in the impact of the driver (a US dollar shock) on the distribution of capital inflows. On the other hand, prior studies have identified periods of extreme capital flows through the threshold method and probit model, which also can effectively capture tail risk, but this method does not quantify the possible severity of extreme capital flows. The quantile regression in this paper can estimate the different impacts of US dollar shocks on the distribution of capital inflows, focusing on the impact on the left tail of the distribution of capital inflows, namely, the impact on the CaR.

### 5.2 The adjustment effect of the exchange rate system under different levels of capital inflows

The regression results in Table 5 show that there is heterogeneity in the average impact of dollar appreciation shocks on cross-border capital inflows under different exchange rate regimes. How does the exchange rate regime work during the different stages of capital inflows? Table 9 reports whether there are differences in the sensitivity of different levels of capital inflows to shocks to the dollar under different exchange rate regimes. First, the regression coefficients of dollar shocks are negative at different quartiles of capital inflows and significant at the 1% level, indicating that a floating exchange rate regime cannot isolate the adverse effects of dollar shocks on the distribution of capital inflows and that dollar shocks still lead to a leftward shift in the distribution of capital inflows and an increase in the risk of lower quartiles of capital inflows even for economies with a floating exchange rate regime. Second, the coefficient on the interaction term between the fixed exchange rate regime and the dollar shock is significantly positive, and the coefficient value is larger at the lower quartile of capital inflows (shown in Columns (1)-(2)). This suggests that fixed exchange rate regimes are more effective at mitigating the sensitivity of capital inflows to dollar shocks in the lower quartile. Thus, the coefficient on the interaction term between the fixed exchange rate regime and the dollar shock is significantly positive at the lowest quartile of mitigation capital, and the coefficient value is larger at the low quartile, indicating that the sensitivity of the different levels of capital inflows to the dollar shock is smaller in an intermediate exchange rate regime than in a floating exchange rate regime. the lower the level of capital inflows is, the better the buffering effect of the intermediate exchange rate regime. Overall, compared to floating exchange rate regimes, fixed and intermediate exchange rate regimes mitigate the risk of leftward capital flows due to dollar shocks, and the lower the level of capital inflows is, the more effective they are at mitigating dollar shocks.

**Table 9:** Panel quantile regressions: Effect of exchange rate regime.

Variable name	(1)	(2)	(3)	(4)	(5)	(6)	(7)	(8)	(9)	(10)	(11)
	5	10	20	30	40	50	60	70	80	90	95
Dusdx	−1.3553***	−1.1379***	−1.0928***	−0.9896***	−0.5503***	−0.5423***	−0.7094***	−0.6355***	−0.6296***	−0.9773***	−0.5619***
	(0.0004)	(0.0000)	(0.0006)	(0.0005)	(0.0002)	(0.0000)	(0.0008)	(0.0002)	(0.0015)	(0.0000)	(0.0002)
Dusdx × reg1	1.0205***	0.6379***	0.8083***	0.7531***	0.3243***	0.3428***	0.5677***	0.3550***	0.2313***	0.6826***	0.2212***
	(0.0005)	(0.0000)	(0.0008)	(0.0004)	(0.0001)	(0.0000)	(0.0007)	(0.0001)	(0.0012)	(0.0001)	(0.0003)
Dusdx × reg2	0.6224***	0.5807***	0.5850***	0.5264***	0.1372***	0.1319***	0.3521***	0.1836***	0.1416***	0.4989***	0.2174***
	(0.0005)	(0.0000)	(0.0007)	(0.0006)	(0.0003)	(0.0001)	(0.0008)	(0.0002)	(0.0016)	(0.0001)	(0.0003)
Controls	Yes	Yes	Yes	Yes	Yes	Yes	Yes	Yes	Yes	Yes	Yes
Observations	2079	2079	2079	2079	2079	2079	2079	2079	2079	2079	2079

Note: ***, ** and *  denote significance at the 1%, 5% and 10% levels, respectively. Standard errors are reported in parentheses.

### 5.3 The moderating role of country characteristics at low quantiles of capital inflows

Considering the interaction between dollar shocks and country structural characteristics, an interaction term between the two is introduced to assess whether the nonlinear effect of dollar shocks on cross-border capital inflows is enhanced through country structural characteristics. Table 8 examines the effect of dollar shocks on the distribution of capital inflows and reveals that the sensitivity of capital flows to dollar shocks is most pronounced in the lower quartiles of capital flows. Therefore, instead of reporting results for each quantile of capital inflows after the inclusion of the interaction term, the focus is on a specific quantile (left tail) and thus on whether country structural characteristics are associated with higher or lower CaR. Table 10 tests whether there are differences in the effects of dollar shocks on the 5% and 10% quartiles of cross-border capital inflows for different country structural characteristics. Columns (1)-(4) show the effects of the degree of financial integration, trade openness, financial market development and capital account openness on capital inflows in the 5% quantile in that order, and Columns (5)-(8) show the effects of the above four factors on capital inflows in the 10% quantile.

**Table 10:** Effect of country structural characteristics: Low quantiles.

	(1)	(2)	(3)	(4)	(5)	(6)	(7)	(8)
Variable name	Fin-Integra	Trade-open	FD	Kao-pen	Fin-Integra	Trade-open	FD	Kao-pen
	5%				10%			
Dusdx	−0.6443***	−0.6682***	−0.2454***	−0.7514***	−0.6888***	−0.5676***	−0.2393***	−0.7231***
	(0.0000)	(0.0002)	(0.0011)	(0.0002)	(0.0018)	(0.0002)	(0.0000)	(0.0008)
Dusdx×lfin	0.0079***				0.0014***			
	(0.0000)				(0.0003)			
Dusdx×ltrade		−0.0127***				−0.1180***		
		(0.0002)				(0.0003)		
Dusdx×lfd			−1.0545***				−0.9918***	
			(0.0024)				(0.0002)	
Dusdx×lkaopen				0.1923***				0.1565***
				(0.0003)				(0.0011)
Controls	Yes	Yes	Yes	Yes	Yes	Yes	Yes	Yes
Observations	2079	2079	2079	2079	2079	2079	2079	2079

Note: The regression results for the other 9 quantiles are not shown and are available on request. ***, ** and *  denote significance at the 1%, 5% and 10% levels, respectively. Standard errors are reported in parentheses.

First, the coefficient on the interaction term between the degree of financial integration and the dollar shock is significantly positive (Columns (1) and (5)), which suggests that the adverse effects of a dollar appreciation shock are mitigated in lower quartiles of capital inflows when a country is more financially integrated. A possible explanation is that the degree of financial integration is one of the factors that global investors consider when making cross-border capital allocations, and the higher the degree of financial integration of a country, the less of incentive investors have to withdraw funds from that country. Thus, a high degree of financial integration in capital-receiving countries can mitigate the adverse effects of a rising dollar index.

Second, Columns (2) and (6) show that the coefficient of the interaction term between trade openness and dollar shocks is negative and significant at the 1% level, indicating that higher trade openness exacerbates the negative impact of dollar shocks on CaR. In general, sudden stops in capital flows tend to lead to a decline in trade credit, with higher trade openness leading to more severe losses when sudden stops in capital inflows occur.

Third, according to the regression results in Columns (3) and (7), the coefficient of the interaction term between financial development and dollar shocks is significantly negative at the 1% level, indicating that an increase in financial development amplifies the negative impact of dollar shocks on CaR. The results in Columns (3) and (7) extend this finding by highlighting that the interaction term between financial development and dollar shocks is significant in lower quartiles of the capital flow distribution. The greater a country’s financial development is, the stronger the reducing effect of a dollar appreciation shock on cross-border capital inflows; moreover, not only does the likelihood of a sudden stop in capital flows increase, but its severity also increases.

Finally, Columns (4) and (8) show that the coefficient on the interaction term between capital account openness and dollar shocks is significantly positive, indicating that higher capital account openness mitigates the sensitivity of capital inflows (CaR) to dollar appreciation shocks at the 5% and 10% quantiles. A possible explanation is that as capital account openness increases, currency market stability rises and transaction costs for cross-border capital flows fall, which in turn mitigates the adverse impact of dollar shocks on lower quantile capital inflows.

## 6. Conclusions

This paper is based on cross-country panel data on 33 emerging economies from 2006q1 to 2021q4. The sensitivity of cross-border capital inflows to dollar appreciation shocks and the dynamic evolution of dollar shocks at different levels of capital inflows are examined to refine the analytical framework of the drivers of cross-border capital flows. The study shows that, first, a dollar appreciation shock leads to a decline in aggregate cross-border capital inflows to emerging economies. Mechanism tests reveal that dollar appreciation affects cross-border capital inflows mainly through domestic and external financial cycle differences, specifically domestic and foreign risk premiums, asset prices, exchange rate volatility and exchange rate expectations channels. Heterogeneity analysis shows that dollar shocks have the least adverse impact on capital inflows under fixed exchange rates. Second, the impact of dollar shocks differs significantly across quantiles of capital inflows, with dollar appreciation shocks leading to a leftward shift in the distribution of capital inflows, increasing the likelihood and severity of sudden stops in capital flows. When more flexible exchange rate regimes are implemented, the negative impact of dollar shocks is exacerbated throughout the distribution of capital inflows, while the moderating effect of fixed and intermediate exchange rate regimes on external shocks can be effective in lower quantiles. Third, the sensitivity of the distribution of cross-border capital inflows to dollar shocks depends on the degree of financial integration, trade openness, financial development, capital account controls, and other country characteristics. Third, the sensitivity of the distribution of cross-border capital inflows to dollar shocks depends on the degree of financial integration, trade openness, financial development, capital account controls, and other country characteristics.

Given those findings, dollar appreciation shocks, as a risk factor for emerging economies, can predict the trend of cross-border capital inflows to emerging economies, and countries should take effective policy measures to curb the adverse effects of dollar volatility.

First, improve the management framework of cross-border capital flows and build a forward-looking monitoring and early warning system for cross-border capital flows. The first task is to incorporate the dollar index into the monitoring and early warning indicator system to prevent the risk of a capital inflow surge due to a dollar depreciation shock and the risk of a sudden capital stop caused by dollar appreciation. The second is to develop a differentiated policy framework based on the sensitivity of different types of capital flows to dollar shocks. Specifically, direct investment has a longer maturity and is relatively less sensitive to changes in external factors such as dollar shocks, while the role of domestic factors such as economic fundamentals, the institutional environment, and trade openness are more critical. Emerging economies can attract foreign investment by strengthening domestic economic fundamentals, creating a favourable institutional environment, and lowering hidden trade barriers. Portfolio investments and other investments have shorter maturities, are more volatile, and are more sensitive to external factors such as dollar shocks. Therefore, the management of short-term capital flows should be strengthened, and an effective data collection, monitoring and early warning mechanism should be established to closely track the dynamic changes in the US dollar index. The third is to improve the exchange rate formation mechanism and moderately increase the flexibility of the exchange rate. It is not the case that a more flexible exchange rate regime is better for the government in implementing exchange rate policy. When faced with a large dollar appreciation shock, increasing the flexibility of the exchange rate regime may be interpreted by the market as a “fragility” signal, which could easily create expectations of exchange rate depreciation and trigger the risk of a sudden stop in capital inflows.

Second, be alert to changes in the dollar index in the process of Fed policy adjustment causing resonant changes in internal and external financial cycle differential indicators, and block the transmission path of the adverse impact of dollar shocks on capital inflows in a timely manner. When financial markets encounter adverse external shocks, the sensitivity of short-term capital flows in emerging economies to USD appreciation shocks is further enhanced under the combined impact of “narrowing interest rate spreads - falling asset spreads - exchange rate depreciation” at home and abroad. Interest rates, asset prices, exchange rates, and exchange rate expectations are important mechanisms through which dollar shocks act on cross-border capital flows. Reasonable policy tools such as monetary policy and foreign exchange intervention are used to smooth fluctuations in indicators of internal and external financial cycle differences to curb large fluctuations in cross-border capital flows.

Third, pay attention to the left-tail risk of cross-border capital flows and include CaR as a reference indicator in the macroeconomic control system. Abnormal cross-border capital flows will raise the level of systemic financial risks and are not conducive to the smooth operation of emerging economies, including China. The government authorities should set a reasonable CaR safety valve, strengthen the dynamic monitoring of short-term cross-border capital flows and stocks, and improve the effectiveness of risk monitoring.

## Supporting information

S1 File Data. (RAR)
